# Insulin use in type II diabetic patients: a predictive of mortality in covid-19 infection

**DOI:** 10.1186/s13098-022-00857-2

**Published:** 2022-06-20

**Authors:** Marc Assaad, Nakisa Hekmat-Joo, Jeff Hosry, Ali Kassem, Ahmad Itani, Loai Dahabra, Ahmad Abou Yassine, Julie Zaidan, Dany El Sayegh

**Affiliations:** 1 Department of Internal Medicine, Staten Island University Hospital, Staten Island, NY 10305 USA; 2 Department of Pulmonary Disease and Critical Care, Staten Island University Hospital, Staten Island, NY 10305 USA; 3 Department of Endocrinology, Staten Island University Hospital, Staten Island, NY 10305 USA

**Keywords:** Covid-19, SARS-CoV-2, Coronavirus, Type II Diabetes mellitus, Insulin Resistance, Insulin, Inflammatory markers, HbA1c, Mechanical ventilation, Invasive ventilation

## Abstract

**Introduction:**

Starting December 2019, the world has been devastated by the rapid spread of coronavirus disease 2019 (Covid-19). Many risk factors have been associated with worse outcomes and death from Covid-19 pneumonia including having diabetes mellitus. To date, it is not clear if all group of diabetics share the same risk of complications with COVID-19 infection. This study aims to compare disease severity and mortality rate in insulin users versus non-insulin users.

**Methods:**

In this retrospective case–control study conducted at the largest health care network in New York state, we included adult, diabetic patients admitted from March 2020 to October 2020 with Covid-19 pneumonia. We compared the baseline characteristics in addition to outcomes of diabetic patients on home insulin (cases) and non-insulin user diabetics (controls). In addition, to determine if home insulin use is associated with an increased mortality, we conducted a cox regression analysis.

**Results:**

We included 696 patients in the study period with a median age of 57 years, interquartile range [IQR] 51–62, and median body mass index 29.9 (IQR: 26–34.7). The majority (476 [68%]) were males. We identified 227 cases (33%) and 469 controls (67%). More cases than controls were hypertensive (74% vs 67%, p = 0.03), on ACE/ARB (50% vs 42%, p = 0.05), and had a hemoglobin A1c > 8.1 (71% vs 44%, p < 0.001). More cases had AKI (52% vs 38%, p < 0.001), however no significant differences were found in intubation rates (26% vs 24%, p = 0.54), detection of pulmonary embolism (4% vs 6%, p = 0.19) or death rate (15% vs 11%, p = 0.22) comparing cases and controls. In a multivariate analysis, we found that home insulin use was independently associated with increased risk of death: Hazard ratio: 1.92, 95% confidence interval (1.13–3.23).

**Conclusion:**

We showed herein that diabetic patients on home insulin with COVID-19 pneumonia, have worse outcomes and increased mortality compared to diabetics on oral antihyperglycemic agents. Close monitoring of insulin-dependent type II diabetic patients is needed in the current pandemic.

## Background

Coronavirus disease 2019 (Covid-19 was reported for the first time in Wuhan, China in December 2019 [1, 2]. The virus belongs to the Coronaviridae family and is a single stranded RNA enveloped virus known as severe acute respiratory syndrome coronavirus 2 SARS-CoV-2 [3]. The mortality rate from Covid-19 is 3.8 % [4] which is lower than genetically similar coronaviruses that caused previous pandemics, while the infectivity rate is high [3].

Identifying patients at risk of severe illness is crucial to prioritize this specific group in terms of treatment and vaccination. Many risk factors have been implicated such as advanced age, male gender, being certain ethnicities such as African American or south Asian, cardiovascular disease, hypertension, chronic obstructive pulmonary disorder, cancer patients, neurocognitive impairment and diabetes mellitus [5, 6].

Diabetes mellitus is a systemic disease, affecting many organs and is associated with insulin resistance in addition to metabolic syndrome which are stated as major risk factors for severe COVID-19 illness. A systematic review has shown that mortality rate in hospitalized patients is higher among diabetic patients compared to non-diabetic [7] making diabetes mellitus a field of interest in our study.

It remains unclear whether all type II diabetic patients share the same risk or those with poorly controlled disease are at higher risk. We sought to evaluate herein if insulin-dependent diabetics have poor outcomes compared to diabetic patients on oral antihyperglycemic.

## Subject and methods

### Study population

We conducted this retrospective case-control analysis in the largest healthcare system in New York state. We included patients admitted from March 2020 to October 2020 to any of the 11 hospitals affiliated with Northwell Health. We only analyzed adult patients (age > 18 years), diagnosed with COVID-19 pneumonia and who had an HbA1c on admission or within 3 months of the diagnosis.

To focus our study on the effect of insulin resistance and inflammatory state in diabetic patients, we excluded patients with concomitant chronic respiratory disease (interstitial lung disease, COPD, asthma), atrial fibrillation, end-Stage Renal Disease (eGFR < 15) heart failure and type I diabetes mellitus. This study was approved by The Northwell Health Institutional Review Board.

### Parameters studied

Electronic medical records were reviewed and data on demographic, age, gender, race, BMI, and smoking status, collected. In addition, we collected information on diabetes treatment such as the use of oral antidiabetic, versus the parenteral insulin at home and the use of ACE inhibitor.

The highest inflammatory marker level was recorded for each of Procalcitonin, ferritin, C-reactive protein, and D-dimer. Treatment received during admission such as steroid, convalescent plasma, tocilizumab, remdesivir, hydroxychloroquine and azithromycin is also detailed.

The primary endpoints studied were the need of mechanical ventilation, thromboembolic events such as deep vein thrombosis (DVT) or pulmonary embolism, acute kidney injury (AKI) defined as an increase in serum creatinine more or equal to 50% from baseline or > 0.3 mg/dl in < 48 h, death, and length of stay. Arterial blood gas for those who required intubation was collected to classify the underlying acute respiratory distress syndrome (ARDS), which is defined as mild when the ratio of arterial oxygen partial pressure (PaO2) to fraction of oxygen in inspired air (FiO2) is more than 200 mmHg, moderate when PaO2/FiO2 is between 100 and 200 mmHg and severe when the ratio is below 100 mmHg.

### Data analysis

We used descriptive statistics to analyze the study population. We used Fisher exact test or Chi-square test to compare categorical variables and Wilcoxon-Mann-Whitney test to compare medians of continuous variables.

To determine the effect of the diabetes and diabetes treatment on COVID disease and response to treatment we compared diabetic patients on home insulin (cases) and non-insulin user diabetics (controls). In addition, to identify predictors of worse outcomes and death, we compared diabetic patients who died versus those who survived COVID-19 infection in a univariate and a multivariate Cox regression model. Results were reported as hazard ratios (HRs), corresponding 95% confidence intervals (CI), and p values. We used the SPSS software program (version 23.0; IBM Corporation, Armonk, NY), all statistical tests were two-sided, and p values < 0.05 were considered statistically significant.

## Results

### Demographics and descriptive analysis

A total of 696 patient were included in the study period The majority were males (476, 68%) The median age of the patients was 57 years (interquartile range [IQR: 51–62]) and the median body mass index (BMI) was 29.9 (IQR 26–34.7). Cohort data collected showed that 170 patients were Caucasian (24%).

Out of the 696 patients 14% were smokers, 69% had pre- existing hypertension, 45% were on angiotensin converting enzyme inhibitor or angiotensin II receptor blockers (ACE-I/ARB), 33% were on home insulin and 51% were on metformin. Throughout their hospital stay, the prevalence of intubation reported was 24% and of AKI was 43%. The mortality rate of this patient population was 12% (Table 1).

**Table 1:** General characteristics

	N = 696
Age, median [IQR]	57 (51–62)
BMI, median [IQR]	29.9 (26–34.7)
Gender	
Male	476 (68%)
Female	220 (32%)
Race	
White	170 (24%)
African American	139 (20%)
Hispanic	132 (19%)
Asian	65 (9%)
Multiracial	190 (27%)
Smoker	96 (14%)
Home Insulin	227 (33%)
Metformin	354 (51%)
ACE inhibitor/ARB	310 (45%)
Hypertension	481 (69%)
Intubation	168 (24%)
Acute kidney injury	298 (43%)
Death	86 (12%)

### Comparison of cases and controls

We identified 227 cases (33%) and 469 controls (67%). More cases than controls were hypertensive (74% vs 67%, p = 0.03), on ACE/ARB (50% vs 42%, p = 0.05), and had a hemoglobin A1c > 8.1 (71% vs 44%, p < 0.001). Cases tend to have higher median D-dimer levels (991 [383–3371] vs 724 [313–2909] p = 0.09) and procalcitonin level (0.41 [0.16–1.42] vs 0.3 [0.11–0.95], p = 0.016). More cases had AKI (52% vs 38%, p < 0.001), however no significant differences were found in intubation rates (26% vs 24%, p = 0.54), detection of pulmonary embolism (4% vs 6%, p = 0.19) or death rate (15% vs 11%, p = 0.22) comparing cases and controls. There was no significant difference regarding length of hospital stay among the two groups (p = 0.22) as shown in Table 2.

**Table 2:** Home insulin use versus no home insulin

	Home insulin N = 227	No home insulin N = 469	p value
ACE inhibitor/ARB	113 (50%)	197 (42%)	0.053
Hypertension	169 (74%)	312 (67%)	0.034
Hb A1c > 8.1%	161 (71%)	205 (44%)	< 0.001
D-dimer	991 [383–3371]	724 [313–2909]	0.092
Procalcitonin	0.41 [ 0.16–1.42]	0.3 [0.11–0.95]	0.016
Acute kidney injury	119 (52%)	179 (38%)	< 0.001
Pulmonary embolism	9 (4%)	28 (6%)	0.269
Intubation	58 (26%)	110 (24%)	0.545
Length of hospital stay, days	8 (4–14)	8 [4–14.5]	0.22
Death	33 (15%)	53 (11%)	0.224

### Predictors of mortality

In a univariate analysis, comparing deceased patients and survivors, male gender (84% vs 66% p = 0.001) and advanced age (60 [54–62] vs 57 [50–62], p = 0.005) were more common among non survivors.

In addition, mortality rate was significantly higher in intubated patients (90% vs 15%, p < 0.001), and patients with moderate to severe ARDS as compared to patients with mild ARDS (p < 0.001). All inflammatory markers including C-reactive protein (CRP), D-dimer, ferritin and procalcitonin were higher among patients who died.

The prevalence of AKI and deep vein thrombosis (DVT) was higher among the deceased group as compared to survivors (86% vs 37%, p = 0.001) and (14% vs 6%, p = 0.003) respectively (Table 3).

**Table 3:** Factors associated with death, Univariate analysis

	Death	No death	p value
Age, median [IQR]	60 [54–62]	57 [50–62]	0.005
Gender, Male	72 (84%)	404 (66%)	0.001
Intubation	77 (90%)	91 (15%)	< 0.001
PaO2/FiO2	141.5 (68.2–223)	285 (191–448)	< 0.001
C- Reactive protein	31.45 (21.8–42.2)	16.3 (8.4–32.3)	< 0.001
D-dimer	4673 (2111–12,447)	639 (310–2460)	< 0.001
Ferritin	2017 (1039–5043)	881 (425–1618)	< 0.001
Procalcitonin	5.25 (0.77–17.68)	0.27 (0.11–0.7)	< 0.001
Acute kidney injury	74 (86%)	401 (66%)	< 0.001
Deep vein thrombosis	12 (14%)	34 (6%)	0.003

**Fig. 1:**
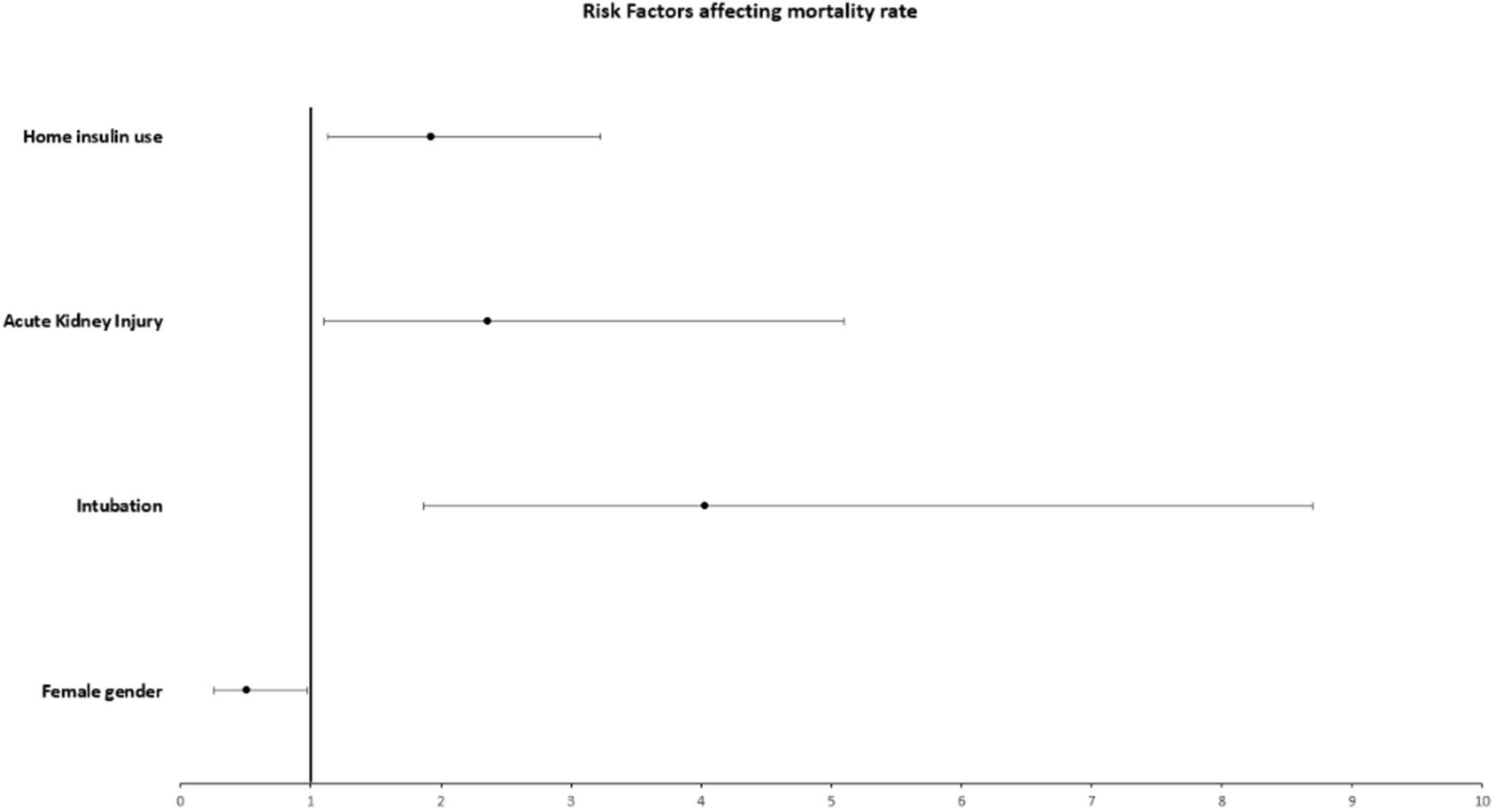
Forest plot illustrating risk factors affecting mortality rate

**Fig. 2:**
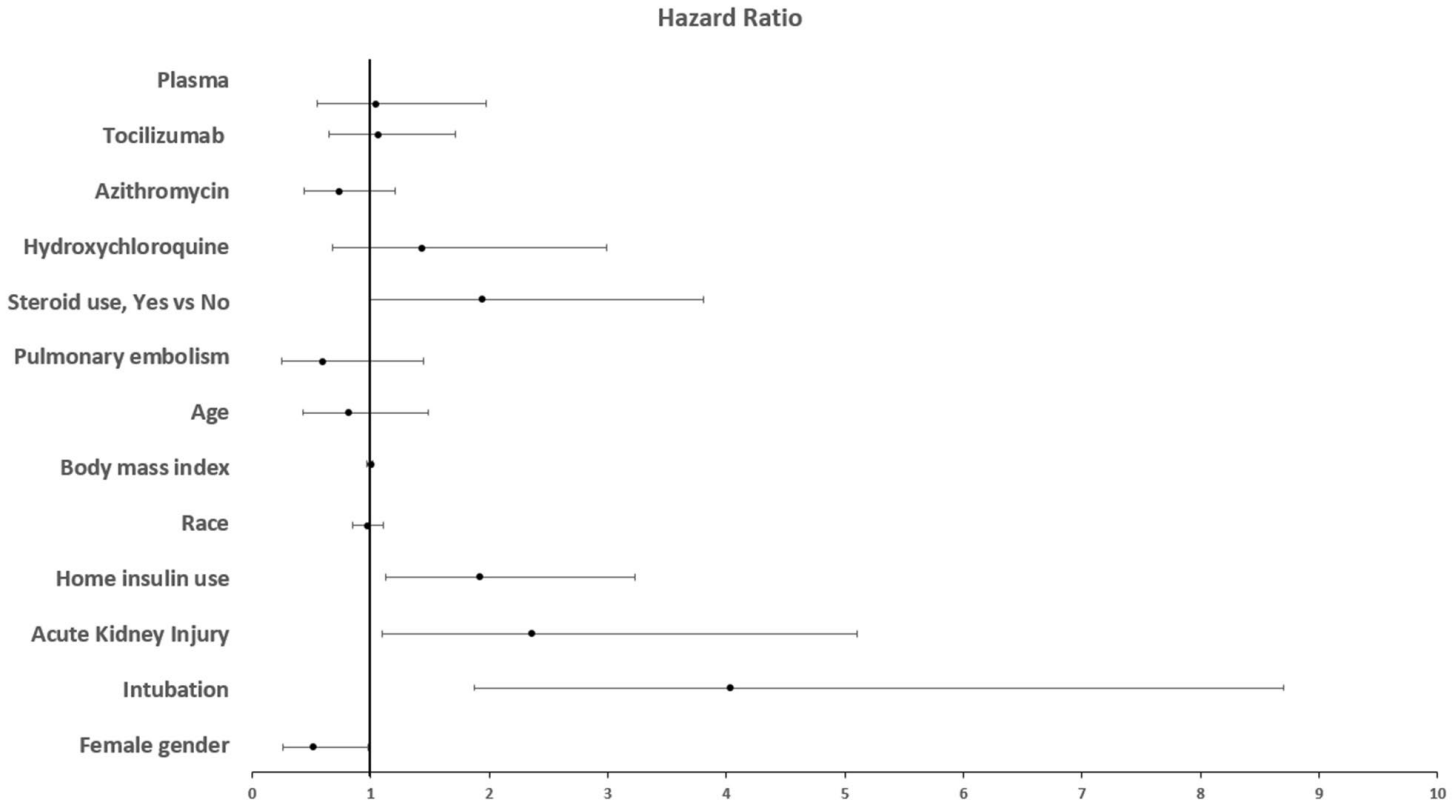
Forest plot illustrating risk factors affecting mortality rate

To determine if home insulin use is associated with an increased mortality, we conducted a cox regression analysis that included many variables (age, race, gender, AKI, BMI, pulmonary embolism, intubation, medical inpatient treatment and insulin use at home). In this multivariable analysis, we found that home insulin use was independently associated with increased risk of death: Hazard ratio: 1.92, 95% confidence interval CI (1.13–3.23), p = 0.014. Female gender was found to be a protective risk factor with HR of 0.51 95% CI (0.26–0.98) p = 0.046. On the opposite side, intubation increases the risk of death by four-fold with HR 4.03, 95% CI (1.87–8.7), p < 0.001 and AKI almost double the risk of death with HR 2.36 95% CI (1.1–5.1), p = 0.028. Increased mortality was not significantly correlated to any of these factors: age, race, BMI, PE, and medical treatment (Table 4) (Figs. 1 and 2).

**Table 4:** Multivariable analysis, or cox regression analysis

	HR	CI	p value
Age	0.81	0.43–1.49	0.486
Race	0.97	0.85–1.11	0.737
BMI	1.001	0.97–1.01	0.942
Acute kidney injury	2.36	1.1–5.1	0.028
Female gender	0.51	0.26–0.98	0.046
Intubation	4.03	1.87–8.7	< 0.001
Home insulin use vs no insulin use	1.923	1.13–3.23	0.014
Steroid use, yes vs no	1.94	0.99–3.81	0.053
Hydroxychloroquine	1.43	0.68–2.99	0.335
Azithromycin	0.73	0.44–1.21	0.231
Tocilizumab	1.06	0.65–1.71	0.812
Plasma	1.04	0.55–1.97	0.892
Pulmonary embolism	0.59	0.245–1.45	0.258

## Discussion

We showed herein that diabetic patients treated with subcutaneous insulin prior to hospital admission had worse outcomes and almost two times higher risk of death compared to diabetics treated with oral antihyperglycemic. Male gender, the need of mechanical ventilation and the development of AKI were associated with higher mortality rate. On the opposite side, female gender was found to be a protective factor. Many factors contribute to this large difference including lifestyle (smoking, drinking, hygiene, safety measures…), immunological difference driven by sex hormones and X chromosome [8] and ACE 2 receptor, which is the port of entry of SARS-CoV-2, that is more expressed in males than in females [9].

Only 14% of the studied sample were found to be smoker. A decent explanation why the percentage of smokers is reduced in our findings, might be the elimination of patients with chronic obstructive pulmonary disease COPD.

Diabetes mellitus is well known to cause immune dysregulation. Insulin resistance has been linked to inflammation through multiple pathways. The adipocyte produces adipokines including interleukin IL-6 and tumor necrosis factor TNF alfa [10] that has been proven to cause insulin resistance [11]. On the same page, acute phase reactants such as C reactive protein (CRP), fibrinogen and white blood cell count were found to be higher in type II diabetic patients [10]. Along with that, hyperglycemia potentiates inflammatory markers favorizing cytokine storm event [12]. A review has shown that diabetes mellitus has been associated with pulmonary endothelial cell dysfunction and activation, placing the vessels at a higher risk of leak and damage with Covid-19 [13].

In our study, higher hemoglobin A1c level did not correlate with an increased risk of death. These results come along with some other findings [14, 15] and contradict on the other hand different results [16, 17]. Many cofounders may have contributed to the non-significant correlation including concomitant anemia, BMI, medication compliance and most importantly sample size.

In a retrospective study dividing patients according to their HbA1c, the mortality rate, inflammation and hypercoagulability were higher with higher A1c [16]. On the other hand a retrospective single-center study conducted on 117 Covid-19 patients with T2DM has failed to show a significant difference between well and poorly controlled diabetes in terms of clinical outcomes with many limitations including sample size and patients’ selection [14]. Another cohort study contradicts these findings by dividing diabetic patients into controlled and non-controlled based on their HbA1c (above or below 8%). Uncontrolled diabetic patients were found to have worse presentation and poorer outcome [18]. Same findings were proven by another cohort study who found that patients with HbA1c > 9% had increased risk of hospitalization and concluded that HbA1c could be a strong predictor for disease severity and outcome [19].

Importantly The CORONADO study performed on a larger sample, failed to determine a positive correlation between A1c and the primary outcome of the illness in a multivariable analysis [17]. However, glycemic control has been proven essential to decrease the length of stay and the mortality rate according to Bruce Bode et al. [20]. Whether hemoglobin A1c level correlate or not with the outcome, it is evident that glycemic control is determining the prognosis of critical patients with Covid-19 [21, 22] and may decrease the expression of ACE2 receptor in the lungs [23].

Our findings demonstrate that the use of home insulin almost doubles the risk of death among diabetic patients who encounter Covid-19 infection. A recently published systematic review and meta- analysis, has shown that diabetic patients treated with insulin had higher admission rate with Covid-19 infection [24]. Increased mortality and increased complication in this group were also reported [24, 15]. However, most of these studies were observational with heterogenous results from five different countries, and the type of diabetes studied was unclear. In addition to that, these studies did not exclude many comorbidities such as chronic obstructive lung disease (COPD) that can be a major cofounder.

Another retrospective study revealed that treatment with insulin alone or in combination with other antidiabetic agent is associated with higher mortality rate in Covid-19 patients than those without insulin treatment and the median length of hospital stay was significantly longer for the insulin group [23]. Yet, baseline characteristics and laboratory findings of the compared groups were mismatched and significantly different.

In parallel to that, an observational study has proven the higher mortality rate in diabetic infected patients with Covid-19 who were using insulin at home and a higher inpatient insulin requirement for those who died from Covid-19 [25]. Higher doses of insulin were found to be associated with higher intubation rate [25]. Major limiting factors were sample size (166) and generalizability.

Multiple factors can explain the difference in mortality between these two groups. One of them is the inflammatory state contributing to hypercoagulable state and organ damage, which are complications associated with higher mortality.

Insulin resistance builds up along with pancreas aging and fibrosis which occur with more advanced disease course of diabetes, influenced also by other factors such as adipocyte accumulation and decreased cellular catabolism. In other words, patients who require insulin, have higher insulin resistance thus higher inflammatory state [10–12]. On the same page, our findings are consistent with higher Procalcitonin and higher D-dimer in patients who were treated with insulin which is found to be a prognostic indicator for poor outcome [26] and higher among deceased patients [27]. Similar results have proved that insulin group and hyperglycemia have more enhanced systemic inflammation and higher inflammatory markers including D-dimer, C-reactive protein, TNF-a and other interleukins [23, 28].

Another reason behind the higher mortality rate in insulin group, is the tendency of this group to develop end organ damage. Most diabetic patients on insulin have chronic kidney disease as microvascular complication of more advanced disease. Patients who have chronic kidney disease are more prone to develop acute kidney injury [21, 22], thus have worse outcome [29].

Furthermore, the hesitance of steroid use initially in insulin-dependent patient, is another cause potentially residing behind the higher mortality in this group. RECOVERY trial has proven that Dexamethasone is effective in reducing mortality in ventilated patients and those who require oxygen [30, 31]. Patients treated with insulin at home received less steroid compared to those who were not on insulin.

## Limitations

Our study has several limitations. Due to its retrospective nature, data availability and multiple biases including selection and confounding bias.

All selected patients were hospitalized and have more severe disease as compared to diabetic patients who were monitored at home and who were not included in our study. Age, body mass index, smoking status, hypertension, and chronic kidney disease all are factors that can potentially distort the measure of the association between the use of home insulin and poorer outcome. More prospective and randomized clinical trials are needed.

Another selection bias was excluding patients who did not have a reported HbA1c within 3 months of hospitalization. Patients who did not have close follow-up and who might have worse disease and poor glycemic control have not been studied. Our analysis has excluded patients with underlying lung disease (COPD), arrythmia, heart failure or end stage renal disease, which affects the generalizability of our findings.

Furthermore, patients with chronic kidney disease were not excluded. It has been proven that preexisting CKD is a contributor to the development of new acute kidney injury or AKI [21, 23] and might not come back to their baseline renal function [32, 33]. Patients with Covid-19 pneumonia who developed AKI had a worse outcome as shown in a retrospective study [29]. Moreover, insulin resistance is more prevalent in patients with chronic kidney disease as described in a systematic review [34]. Thus, enrolled patient with chronic kidney disease, have higher insulin resistance, higher inflammatory state, and higher incidence of AKI which might have contributed to a higher mortality and morbidity rate in the group of patients who were treated with insulin at home. Our analysis does not also stratify patients based on their CKD-stage.

Glycemic level along with inpatient insulin requirements are not reported in our analysis which is also a serious limitation. Poor glycemic control during the hospital course might also affect the mortality and the outcome. Thus, further prospective studies including these factors are needed.

## Conclusion

In conclusion, we encountered higher mortality rate and intubation in diabetic infected patients who were treated with subcutaneous insulin at home, as compared to those who were on oral regimen. The importance of the study resides in highlighting the mechanism behind the inflammatory state in this specific population who is at higher risk of death from Covid-19. Stratifying these patients according to their A1c, BMI and treatment is a potential scoring system to predict their outcome. Glycemic control seems to be crucial in decreasing the chronic inflammatory state, thus in protecting diabetic patients not only from Covid-19, but also from any pandemic that may possibly arise in the future.

## Data Availability

Not applicable.
