# Correction to: Insulin use in type 2 diabetic patients: a predictive of mortality in covid-19

**DOI:** 10.1186/s13098-022-00866-1

**Published:** 2022-07-15

**Authors:** Marc Assaad, Nakisa Hekmat-Joo, Jef Hosry, Ali Kassem, Ahmad Itani, Loai Dahabra, Ahmad Abou Yassine, Julie Zaidan, Dany El Sayegh

**Affiliations:** 1 Department of Internal Medicine, Staten Island University Hospital, Staten Island, NY 10305 USA; 2 Department of Pulmonary Disease and Critical Care, Staten Island University Hospital, Staten Island, NY 10305 USA; 3 Department of Endocrinology, Staten Island University Hospital, Staten Island, NY 10305 USA

## Correction to: Diabetology & Metabolic Syndrome (2022) 14:85 10.1186/s13098-022-00857-2

Following publication of the original article [1], the publisher identified an error in article title. The correct title is given below.

- Insulin use in type 2 diabetic patients: a predictive of mortality in covid-19

The original article has been [1] revised.
